# Bayes-LQAS: classifying the prevalence of global acute malnutrition

**DOI:** 10.1186/1742-7622-7-3

**Published:** 2010-06-09

**Authors:** Casey Olives, Marcello Pagano

**Affiliations:** 1 Harvard School of Public Health, Boston, MA, 02115, USA

## Abstract

Lot Quality Assurance Sampling (LQAS) applications in health have generally relied on frequentist interpretations for statistical validity. Yet health professionals often seek statements about the probability distribution of unknown parameters to answer questions of interest. The frequentist paradigm does not pretend to yield such information, although a Bayesian formulation might. This is the source of an error made in a recent paper published in this journal. Many applications lend themselves to a Bayesian treatment, and would benefit from such considerations in their design. We discuss Bayes-LQAS (B-LQAS), which allows for incorporation of prior information into the LQAS classification procedure, and thus shows how to correct the aforementioned error. Further, we pay special attention to the formulation of Bayes Operating Characteristic Curves and the use of prior information to improve survey designs. As a motivating example, we discuss the classification of Global Acute Malnutrition prevalence and draw parallels between the Bayes and classical classifications schemes. We also illustrate the impact of informative and non-informative priors on the survey design. Results indicate that using a Bayesian approach allows the incorporation of expert information and/or historical data and is thus potentially a valuable tool for making accurate and precise classifications.

## Introduction

The frequentist approach to statistical inference assumes that a parameter of interest is a fixed and unobservable quantity. The goal is to make inference about this fixed value, given an assumed sampling distribution of the data. For example, one might estimate the prevalence of disease in a population and calculate a confidence interval about the estimate to reflect the statistical uncertainty associated with the estimation; or test a hypothesis about the value of the prevalence and report a p-value to determine significance. The attributes of these methods are judged *a priori*, or before observing any data. For example, a 95% confidence interval will capture the true parameter value on average 95% of the time. Similarly, a hypothesis test is designed to a certain power function, which determines the potential errors. Yet, once the data have been observed, *a posteriori *the probability that the true parameter lies within that interval is zero or one and the result of the hypothesis test is correct or not- and, unfortunately, we do not know which.

It is common for novice statisticians to make such statements as "the probability that the prevalence lies within the confidence interval is 95%", which is, of course, incorrect in the frequentist framework. Indeed, such statements would be attractive and desirable, if only they were correct. There is a vehicle for making probability statements about distributions of unknown parameters: Bayesian inference [1]. In the Bayesian framework, the unknown parameter is treated not as a constant, but as a random quantity, which varies according to some probability distribution. At the core of this theory is Bayes theorem, which states that for two events, *A *and B, where *Pr*(*B*) > 0, the conditional probability

In practice, *A *and *B *are replaced by the unobservable parameters, *ϕ*, and the observable data, *X*, respectively. Then the expression becomes

The relevant pieces of this expression are the likelihood, *Pr*(*X*|*ϕ*) and the prior distribution, *Pr*(*ϕ*), which, together with *Pr(X*), yield the posterior distribution *Pr*(*ϕ*|*X*). Both ingredients must be specified for valid conclusions in this context. The posterior distribution probabilistically describes the behavior of the unknown parameter given the prior and observed data, and serves as the basis for Bayesian analysis. Certain applications naturally lend themselves to a Bayesian approach. Consider monitoring the prevalence of acute malnutrition amongst children 6-59 months of age and within a particular area. At any given time, there may be a true value of the prevalence of acute malnutrition (i.e. the number of children acutely malnourished in the area divided by the total number of children in the area). However, if one were to consider the prevalence over a six month period, this value would fluctuate as children age, thereby entering or exiting the cohort, or their nutritional statuses change. Thus, it may be more realistic to model the prevalence of malnutrition as a random quantity over time rather than a fixed quantity.

Deitchler *et al *found Lot Quality Assurance Sampling (LQAS) to be a useful tool to monitor the prevalence of Global Acute Malnutrition, defined as Weight-for-Height-Z-score < -2 standard deviations, in emergency situations [2,3]. With any LQAS application, the goal is to classify the population prevalence as above or below predefined thresholds by comparing the number of failures in a random sample to a specific decision rule. For example, one might be interested in determining with a high degree of confidence whether the prevalence of acute malnutrition is greater than 10% in a given population of children less than 5 years of age. To accomplish this goal, randomly sample 200 children from within the population. If more than thirteen children exhibit signs of acute malnourishment, then classify the prevalence of acute malnutrition as greater than 10% in that population. The choice of sample size and decision rule determine the degree to which one can rely on this classification. The LQAS procedure is discussed in depth in the next section. Recently, Bilukha [4] and Bilukha and Blanton [5] substantially criticized these designs in this journal. The main problem with their criticism is exemplified in Bilukha and Blanton's suggestion to use as an alternative measure of risk, "the statistical probability of the true population value's exceeding the threshold," conditional on the number of malnourished children in a sample [5]. When the prevalence is treated as a constant, as it is in the model in their paper, this is a measure with little meaning. The authors fall short of specifying the necessary assumptions to make what is clearly a Bayesian statement; namely, no mention is made of the prior distribution. The reader is left to assume that the authors did not consider this aspect in their calculations.

It is attractive to have the ability to make a probability statement about the prevalence of malnutrition, even though it does require that more structure be imposed on the model. The Deitchler *et al *LQAS designs serve as a natural place to begin this investigation. Classical LQAS has generally relied on frequentist statistical principles, particularly in its application in health (see [6] for over 800 examples, all of which take a frequentist approach). Further, nowhere in the health literature have Bayesian considerations been incorporated into the LQAS procedure, to the best of our knowledge. Yet, Bayes-LQAS (B-LQAS) is well-established in the industrial literature, where it is known as Bayesian Acceptance Sampling (see [7], and references therein). As early as the 1960's, Brush examined classical and Bayesian risks for a variety of sampling plans [7]. Brush, and Sharma and Bhutani [8], emphasize the importance of examining both the classical and Bayes risks when deciding upon a sampling design. Fan [9] and Sheng and Fan [10] consider B-LQAS for binomial testing and outline an approach to choosing a prior based on historical data using an empirical Bayes approach.

More recently, Moskowitz considers B-LQAS under quadratic and step-loss functions [11]. Fitzgerald looks at B-LQAS plans under an assumed mixture prior [12], and he bases B-LQAS designs on Bayes OC curves and average OC curves. These curves were first introduced by Easterling [13]. Now, B-LQAS has made a transition into the economics and operations research literature. For example, in 1996 Lattimore *et al *used B-LQAS to monitor drug use in Illinois. In that application, the sampling plan was determined by minimizing expected cost, as advocated by Moskowitz *et al *[14,15].

In this paper, we discuss the potential benefits of using B-LQAS in health applications. As a running example, we discuss an application to acute malnutrition, motivated by LQAS designs proposed by Deitchler *et al *to classify the prevalence of malnutrition [2,3]. We show how to approach the classification problem from a Bayesian perspective, show some of the advantages of this approach, and discuss the parallels between the classical and Bayesian approach.

## Discussion

### A Brief Review of LQAS and B-LQAS

#### LQAS

Classical LQAS is primarily a classification procedure [16]. In its simplest form, the goal is to classify the unknown prevalence of a binary indicator as greater than or equal to some critical threshold, p*, or less than this threshold. To do so, the number of cases, *Y*, in a simple random sample of size *n *are compared to a predefined *decision rule, d*. If fewer than *d *cases are observed, then the prevalence is classified as low (p < p*). Otherwise, it is classified as high (p ≥ p*). The sample size and decision rule are chosen to achieve error probabilities of *α *and *β*, where the former is the maximum acceptable probability of a false negative (or a false *low*) over some range of prevalences and the latter is the maximum acceptable probability of a false positive (or false *high*) over some other range of prevalences.

To define these ranges, LQAS uses upper and lower thresholds, *p*_*U *_and *p*_*L*_. The sample size and decision rule are chosen so that the probability of a false negative when the true prevalence is greater than or equal to *p*_*U *_is less than or equal to *α*. Likewise, the probability of a false positive when the true prevalence is less than or equal to *p*_*L *_is less than or equal to *β*. In the industrial literature, these errors are referred to as the consumer and producer risks. In health applications, generally *p*_*U *_is chosen to be equal to the critical threshold p* and *p*_*L *_is chosen to reflect the desired detectable deviation from that threshold [16]. However, some have suggested using p* = *p*_*L *_[17], which might be appropriate depending on the application. When deciding on how to implement the procedure, it is important that the investigator keep in mind what *p *represents, particularly if *p *is the prevalence of an undesirable outcome.

**Figure 1:**
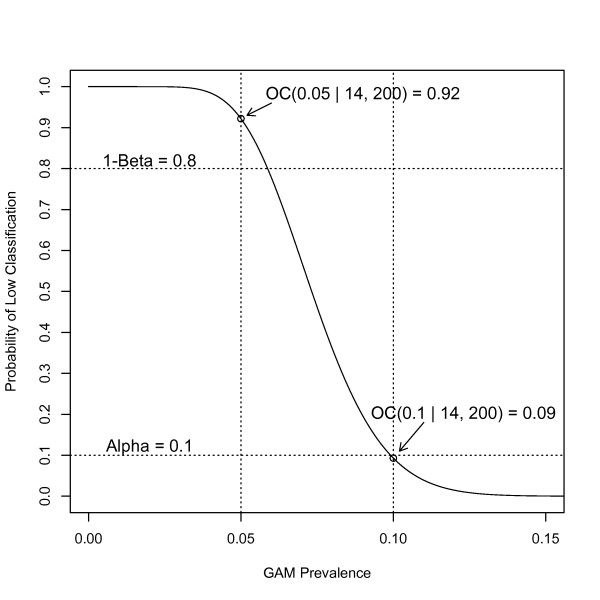
**Classical Operating Characteristic Curve with sample size *n *= 200, decision rule *d *= 14, upper threshold *p*_*U *_= 0.10, lower threshold *p*_*L *_= 0.05, and maximum tolerated errors *α *= 0.10 and *β *= 0.20**.

The Operating Characteristic (OC) Curve completely summarizes any LQAS design. For a given value of *p*, this is defined as(1)

where *Y *is assumed to be binomially distributed with parameters *n *and *p*. Plotting this quantity for the entire range of *p *yields the desired curve. A satisfactory LQAS design will have the following properties:

For example, in Figure 1, we see an OC curve with *n *= 200 and *d *= 14, which corresponds to the LQAS design used by Deitchler *et al *[2,3] to classify the prevalence of malnutrition with a critical threshold p* = 0.10 and with *p*_*U *_= 0.10 and *p*_*L *_= 0.05. In that application, *α *was set to 0.10 and *β *to 0.20. We see that the OC curve is less than *α *= 0.10 at the upper threshold and greater than 1 - *β *= 0.80 at the lower threshold, and thus meets the design requirements.

#### B-LQAS

B-LQAS is similar to classical LQAS in that the final goal is decide whether to classify the prevalence as greater than or equal to some threshold p* , or less than this threshold, so that appropriate action be taken. However, in the Bayesian context, we allow a prior distribution of the parameter, *p*, to be part of the analysis. As with classical LQAS, we choose the sample size, *n*, and the decision rule, *d*, to achieve certain criteria when performing the classification *before *observing the data. In contrast to classical LQAS, these criteria are based on posterior properties of our decision, or what we believe *after *observing the data. For example, it might be important to know what is the probability we have made the correct decision, or classification, and we have the choice of two probabilities, depending on which decision we make.

**Figure 2:**
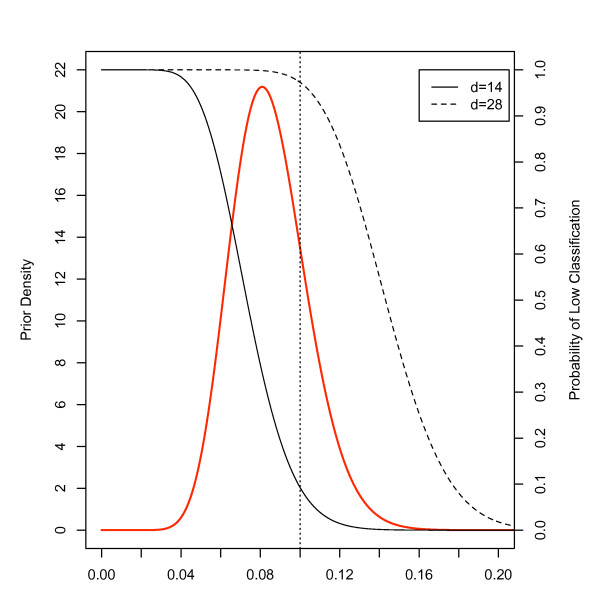
Hypothetical prior distribution of acute malnutrition with mean 8.5% (**) and candidate OC curves for LQAS classification with *d *= 14 (-) and *d *= 28 (--).

To get a better understanding of the intuition behind this approach, consider Figure 2, where we have plotted a hypothetical prior distribution of the prevalence of malnutrition. This distribution has mean 8.5% with 77% of its mass between 5% and 10%. Overlaid on this plot are two OC curves. The solid curve corresponds to a classical OC curve with *d *= 14, or the decision rule that we would choose using classical considerations. When we consider the prior distribution, we might argue that it makes less sense to use this decision rule, which prioritizes error above 10% and below 5%, since we seldom expect to see a prevalence as high or as low. The dashed line corresponds to a classical OC curve with *d *= 28. With the chosen prior, this appears to be a better design as it prioritizes the region of largest prior mass when choosing a decision rule. That is, the design prioritizes correct classification of prevalences which are *most likely *given our prior beliefs. Hence, prior beliefs about the parameter of interest should play a vital role in determining an appropriate design, and in explaining its properties.

For the sake of illustration, in this paper we assume the conjugate Beta prior on the prevalence, *p *~ *Beta*(*a*, *b*), to demonstrate the effect on the OC curves. That is, we let the prior distribution, *π*(*p*), take the structural form

where *a*, *b *> 0 and *B*(*a*, *b*) is the beta function [18]. The Beta provides a rich family of distributions, allowing for a range of flexible prior shapes. Further, there is some precedence for its use [10]. The parameters *a *and *b *control the shape of the prior distribution. To aid interpretation, we might think about *a *and *b *as the prior number of successes and failures, respectively. Therefore, a large value of *b *relative to *a *yields a distribution skewed to the left.

**Figure 3:**
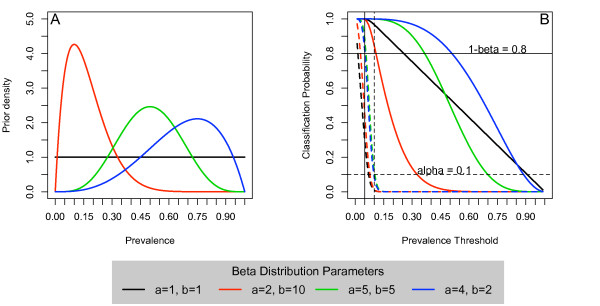
**(A) Various Beta distributions to describe a range of potential prior beliefs**. We assume that *p *~ *Beta*(*a*, *b*). (**B**) Bayes OC Curves assuming *n *= 200 and *d *= 14. The dashed lines (---) represent 1 - *BOC*_*F *_and the solid lines (-) represent 1 - *BOC*_*P*_.

For pedagogical reasons, we choose these parameters to reflect a variety of potential prior beliefs (see Figure 3A). For example, when *a *= 1 and *b *= 1, the prior is completely flat, which might correspond to a lack of prior knowledge or possibly prior indifference. When *a *= 2 and *b *= 10, this corresponds to a prior density with most of its mass below 30%, which is a realistic assumption as malnutrition prevalence is rarely as high as 30%. For example, in the CE-DAT global database of over 1400 malnutrition surveys conducted in emergency situations, only 59 reported a prevalence as high as 30% in children 6-59 months [19]. However, we also look at the case where *a *= 4 and *b *= 2 and where *a *= *b *= 5, reflecting a prior belief that the prevalence is in fact quite high, even though this condition is probably quite unlikely in the present context. The properties of a B-LQAS design can once again be formalized in the OC curves, although we now focus our attention on the Bayes OC curves. A key difference between classical LQAS and B-LQAS is the reliance on not one, but two curves to determine appropriate designs, since we need to condition on either a high or low classification. In this paper, we define the following Bayes OC curves(2)(3)

where the event *Pass *= {*Y *≥ *d*} and *Fail *= {*Y *<*d*},where *Y *is the number of "successes" in a sample of size *n*. Plotting (2) and (3) as a function of *x *yields the desired curves. We can write

where *π*(*p*) is the prior distribution of *p *and *f *(*y*|*p*) is the sampling distribution of *Y *given the parameter, *p*. As a result, the Bayes OC curves are less straightforward to calculate than the classical OC curves, as some integration over the unknown parameter is required. In some cases, these integrals can be analytically intractable, in which case one would have to appeal to numerical methods to evaluate the expressions. In any single application we ultimately take only a single action, but we need to consider both Bayes OC curves. Note that in the case of malnutrition, if *Y *≥ *d*, this indicates a high burden of malnutrition. Therefore, the use of the word *Pass *is not instinctual. However, we might think of *Pass *as "qualifying for humanitarian aid" to facilitate the interpretation. We continue with this notation to provide a unified framework.

The interpretation of each of these curves allows us to make probabilistic statements about the parameter of interest, given the results of our diagnostic procedure; such as statements like those made by Bilukha and Blanton [5], which in their context are incorrect. Intuitively, it would be desireable to control for the probability that the prevalence is low when we say it is high (or declare a *Pass*), for example, and vice-versa. Using the Bayes OC curves, we can choose *n *and *d *so that the Bayes classification errors are controlled. For an analogue to classical LQAS, we can enforce the following:(4)(5)

where *p*_*U *_= p* and *p*_*L *_is some lower critical threshold. However, it is also possible to choose *p*_*L *_= *p*_*U *_= p*, which might be more appealing to some practitioners. This latter case is discussed at length in the context of Phase II clinical trials by Wang *et al *[20]. Ultimately, the choice depend on the application and the priorities of the investigators. We discuss this issue further in the next section.

In Figure 3B, we see the Bayes OC curve plotted as a function of the prevalence threshold *(x *in equations (2) and (3)) with *n *= 200 and *d *= 14. When *α *= 0.10 and *β *= 0.20, we see that the constraints posed in (4) and (5) for *p*_*U *_= 0.10 and *p*_*L *_= 0.05 are met for all considered priors. That is,

Hence, when we choose *d *= 14, which corresponds to the classical solution, we achieve reasonable Bayesian properties as well.

Note that when we let *p*_*L *_= *p*_*U *_= 0.10, the error at the upper and lower thresholds increases slightly as compared to the case when *p*_*U *_= 0.10 and *p*_*L *_= 0.05 for the considered priors. That is, 1 - *BOC_P _*< ( *p *= 0.10 | *n*, *d*) decreases from essentially one, to just over 0.80, in the case when *a *= 2 and *b *= 10, which is still within the design constraints. This is important, however, as it highlights the tradeoff between classification precision and accuracy in these applications. Namely, by classifying as above *p*_*U *_= p* or below *p*_*L*_, we are asking for slightly imprecise results, but doing so with high accuracy. However, if we classify as above or below *p*_*U *_= p*, we are asking for highly precise results, but doing so with less accuracy. We revisit this notion in more depth below.

### Maximizing the Figure of Merit

In general, the above outlined approach is feasible yet time consuming, as it might require an investigator to look at a range of sample sizes and decision rules to arrive at a given design. A more automated design selection is achieved by using the following *Figure of Merit *(FOM),(6)

and for a given sample size, one might choose the decision rule to maximize this quantity. In the decision theoretic literature, this quantity is known as the Bayes Risk of a zero-one utility (negative loss) function [1]. When *p*_*U *_= *p*_*L*_, the FOM of a given design is the average probability of correct classification given a prior *π*(*p*). When *p*_*L *_<*p*_*U*_, dividing the above quantity by a factor of 1 - *Pr*(*p*_*L *_<*p *<*p*_*U*_) yields the average probability of correct classification of priority locales. This scaling of the FOM does not affect the maximization, but can help with interpreting the result. Interestingly, (6) can be rewritten as

which shows that an optimal design is one that maximizes a weighted average of the Bayes OC curves, where the weights are the marginal probabilities of passing and failing the procedure. The marginal distribution of *Y *is also referred to as the *prior predictive *distribution. That is, the predictive distribution of *Y *given only our prior assumptions. Hence, we weight more heavily the Bayes OC curve which has the greater prior predictive probability of occurring.

**Figure 4:**
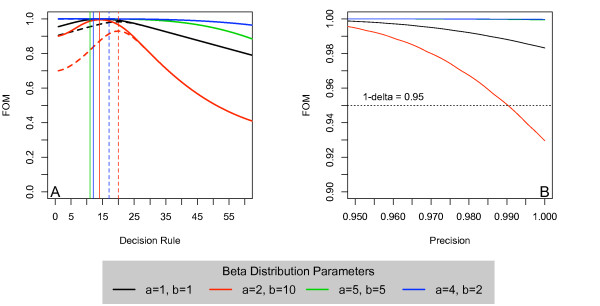
**(A) The Figure of Merit plotted as a function of *d *where *p *~ *Beta*(*a*, *b*) and assuming *n *= 200, *p*_*U *_= 0.1 and both *p*_*L *_= 0.05 (-) and *p*_*L *_= 0.1(---)**. Solid vertical lines indicate the maximum FOM when *p*_*L *_= 0.05 and dashed vertical lines indicate maximum FOM when *p*_*L *_= 0.10. (**B**) Maximum FOM as a function of the precision with *p*_*U *_= 0.1 and *p*_*L *_varying from 0.05 to 0.1 for various assumed Beta priors.

Continuing with our example, Figure 4A shows the plot of the FOM as a function of *d *for the situations when *n *= 200, *p*_*U *_= 0.10, *p*_*L *_= 0.05 and 0.10, and four prior distributions. When *p*_*L *_= 0.05, the optimal decision rule hovers around *d *= 14, or the same as the classical LQAS solution. Yet, it is important to note that both when *a *= 5, *b *= 5 and *a *= 4, *b *= 2, the optimal decision rules are less than 14. Further, even though the curves are very nearly flat in the displayed range of rules, these are true maxima due to the fact that the prior mass below *p*_*L *_is non-zero. Scaling the maximum FOM appropriately reveals that the average probability of correct classification of priority locales is close to 100%, indicating the appropriateness of the design for detecting extremes (i.e. areas where *p *≤ *p*_*L *_or *p *≥ *p*_*U*_).

When *p*_*L *_= *p*_*U *_= 0.10, the maximum FOM decreases, albeit slightly, and the optimal decision rule increases to *d *= 17 or *d *= 20, depending on the prior. Therefore, if our prior belief is that the malnutrition prevalence is low, we require a *greater *number of malnourished children in our sample to be convinced otherwise. But if we believe that the malnutrition prevalence is high, we will need fewer malnourished children in our sample to be convinced that the prevalence is indeed low, and thus possibly triggering an earlier intervention. This is a consequence of incorporating prior information into our analysis. In either case, it is important to realize that the optimal design does a good job of classifying areas. That is, in both cases, the maximum FOM is greater than or equal to 90%. Therefore, with a sample of size *n *= 200, the probability that we correctly classify an area is greater than or equal to at least 0.90.

It is interesting to note that when *a *= 1 and *b *= 1, the in difference prior, the optimal decision rule is equal to 20, or np*. Both Bilukha and Blanton [5] and Rhoda *et al *[17] have suggested using *d *≈ np* for the decision rule in the classical setting. The use of a flat prior with *p*_*L *_= *p*_*U *_= p* gives a Bayesian justification for such a choice, although the use of a flat prior does not ordinarily make sense for this application, as it is uncommon for the prevalence of acute malnutrition to reach as high as 30%, much less 80% or 90% [19]. We discuss this in more depth in the following sections.

### Balancing Accuracy and Precision

The FOM measures the overall *accuracy *of the B-LQAS procedure, and it is attractive to constrain our procedure to achieve at least a minimum FOM. Namely, we constrain the FOM so that(7)

where the parameter *δ *controls the overall level of accuracy in the procedure. This might be considered a more appealing design metric than *α *and *β*. Of course, when *p_L _*= *p_U_*, constraints (4) and (5) imply

Hence, in this special case, the optimal design which meets (7) might be chosen as that design for which a weighted average of the producer and consumer risks, weighted according to the prior belief of passing or failing, is greater than or equal to 1 - *δ*. If *α *= *β*, then we have that *α *= *β *= *δ*, further simplifying the parameterization.

The precision demanded of the procedure impacts the accuracy. That is, the choice of *p*_*U *_and *p*_*L *_affects the properties of the design. Formally, define the *precision *as 1-|*p*_*U*_-*p*_*L*_|.When *p*_*U *_= *p*_*L*_, the precision is equal to one. But as *p*_*L *_deviates from *p*_*U *_, the precision decreases. Indeed, when at their maximal difference, the precision is zero. In our example, *p*_*U *_= 0.10 and *p*_*L *_ranges from 0.05 to 0.10, so that the precision ranges from 0.95 to 1.00.

In Figure 4B, we plot the maximum average probability of correct classification of priority locales (or the appropriately scaled FOM) as a function of the precision, fixing *p_U _*= 0.10 and allowing *p*_*L *_to vary from 0.05 to 0.10. Therefore, when the precision is equal to 0.95, this corresponds to *p*_*L *_= 0.05 and *p*_*U *_= 0.10. When the precision is equal to one, then *p*_*L *_= *p*_*U *_= 0.10. Assume that we want a design that achieves an overall accuracy of 0.95 (1 - *δ *= 0.95). We see that for three of the four considered priors, the maximum FOM is well above 0.95 for all considered precisions, and therefore we should on average correctly classify over 95% of locales with these procedures. However, for the situation when *a *= 2 and *b *= 10, which is likely the more realistic prior for this application, the maximum FOM drops below 0.95 as *p*_*L *_approaches *p*_*U *_, or the precision approaches one. Hence, it is not always possible to achieve the desired level of accuracy for all precisions, short of increasing the sample size; illustrating the trade of between the two.

## Conclusion

In this paper, we describe the basic framework for performing Bayes-LQAS, using as an example an application to acute malnutrition. The benefits of using such a method include the ability to incorporate mild or strong prior beliefs about the underlying distribution, based either on historical data or even expert opinion, and the provision of a principled framework for accumulating data, which can be used in subsequent surveys to inform decision making.

Further, B-LQAS allows for the investigator to make probabilistic statements about the prevalence itself, given the outcome of the classification procedure, which classical LQAS does not. Using the FOM allows for the selection of a design with optimal *a priori *probabilities of correct classification.

We also see the inherent tradeoff between accuracy and precision. This tradeoff is not unique to the Bayesian framework, of course. Indeed, it is this very tradeoff that motivates the use of upper and lower thresholds to evaluate error in the classical LQAS framework. This is due to the fact that it is impossible to make completely accurate classifications for all values of p, barring an infinite sample or a complete census. An important aspect of this tool which we have not discussed is its potential as a routine tool for monitoring population health. Indeed, the Figure of Merit approach can be easily adapted to incorporate historical or routine data. The above formulation is simple by construction, as we wish only to illustrate the potential of B-LQAS. More complex modeling is required to exploit the full utility of this method for monitoring health programs over time. For use with panel data, or repeated cross-sectional surveys over regular intervals, the extension of the above method needs investigating.

Clearly, the choice of prior distribution is an important element of B-LQAS. One alternative to complete specification of the prior is to let the data influence its shape via empirical Bayes procedures (see [21], pg. 122-126 for further discussion). Regardless, the prior can have minor or major influence on the chosen design, depending on the situation. In the example we present, the sample size for the survey is relatively large. However, it is not uncommon to use much smaller sample sizes when performing LQAS(*n *= 19, e.g.) [16]. In this case, the prior distribution will impact the choice of design more heavily. Most importantly, the prior should accurately reflect prior beliefs and should not be chosen to subvert the classification procedure.

## Competing interests

The authors declare that they have no competing interests.

## Authors' contributions

CO and MP contributed jointly to this research. All authors read and approved the final manuscript
